# Supplementary material for: Confirmation of Statin and Fibrate Use from Small-Volume Archived Plasma Samples by High-Throughput LC-MS/MS Method
Source: Int J Mol Sci. 2023 Apr 27;24(9):7931. doi: 10.3390/ijms24097931 (PMC10178340; doi:10.3390/ijms24097931)
Supplement: Supplementary file 1 [file ijms-24-07931-s001.zip › Supplementary Figures.pdf]

Supplementary material

Title: Confirmation of statin and fibrate use from small-volume archived plasma samples by high-throughput LC-MS/MS method.

Journal name: International Journal of Molecular Sciences

Authors: Jennifer D. Kusovschi[1], Anna A. Ivanova[1], Michael S. Gardner[1], Robert W. McGarrah III[2], William E. Kraus[2], Zsuszanna Kuklenyik[1]*, James L. Pirkle[1], John R Barr[1]*

[1]Division of Laboratory Sciences, National Center for Environmental Health, Centers for Disease Control and Prevention, Atlanta, GA 30341

[2]Duke Molecular Physiology Institute, Duke University School of Medicine, Durham, NC, USA

*Corresponding Authors:

John R. Barr, Ph. D, Clinical Chemistry Branch, Division of Laboratory Sciences, National Center for Environmental Health, Centers for Disease Control and Prevention, Atlanta, GA 30341; E-Mail: jbb0@cdc.gov; Phone: +1-770-488-7848; Fax: +1-770-488-0509.

Zsuszanna Kuklenyik, Clinical Chemistry Branch, Division of Laboratory Sciences, National Center for Environmental Health, Centers for Disease Control and Prevention, Atlanta, GA 30341; E-Mail: czk9@cdc.gov; Phone: +1-770-488-7923.

Target	Precursor Nominal Mass (Da) and Structure	Product Nominal Mass (Da) and Proposed Structure
2-OH-Atorvastatin[1]	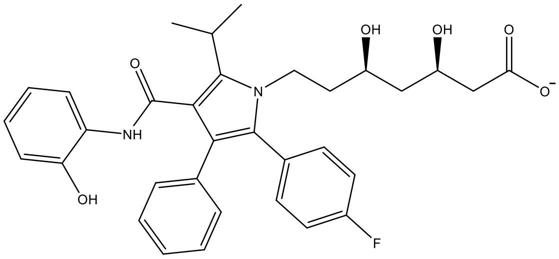 573	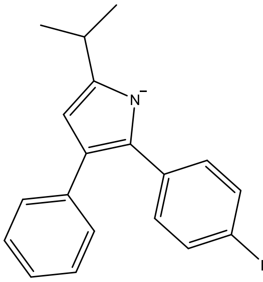 278
4-OH-Atorvastatin[1]	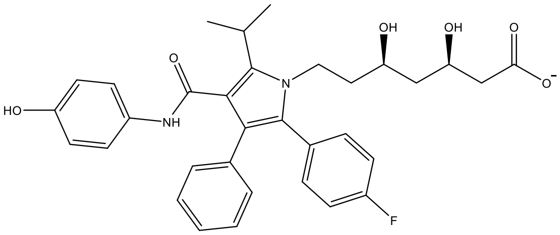 573	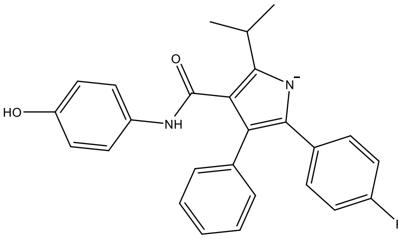 413
Atorvastatin[2]	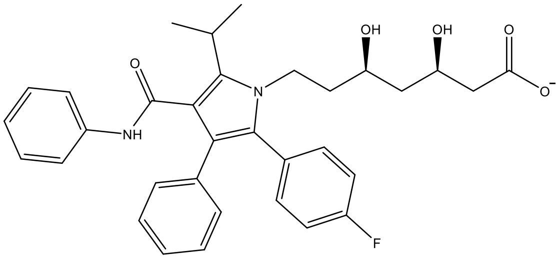 557	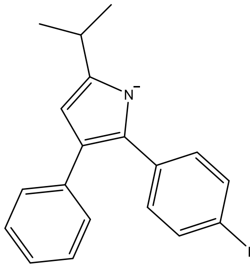 278
Clofibric Acid[3]	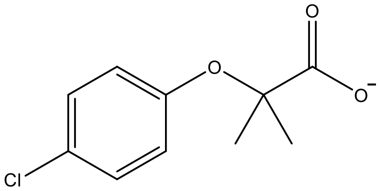 213	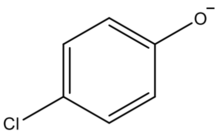 127
Fenofibric Acid[3]	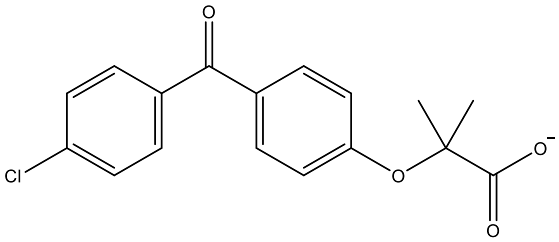 317	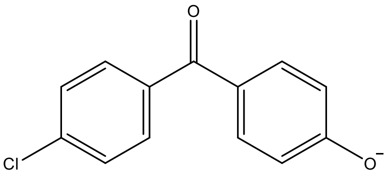 231

Fluvastatin[3]	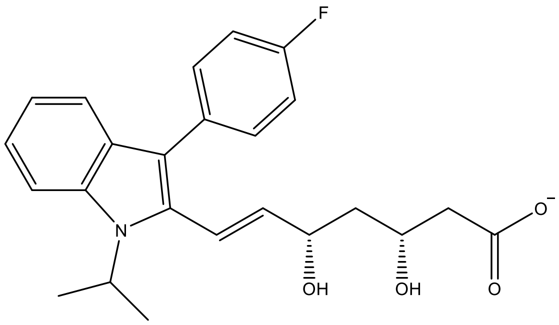<br>410	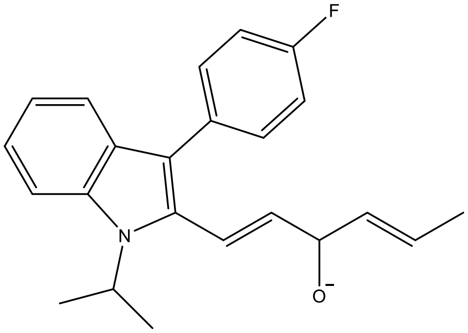<br>348
Gemfibrozil[3]	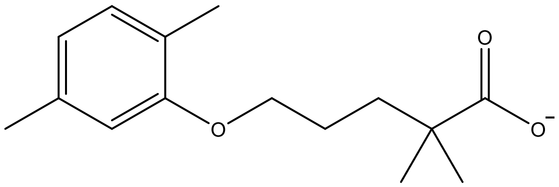<br>249	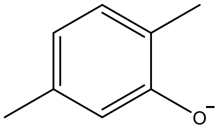<br>121
Lovastatin Acid	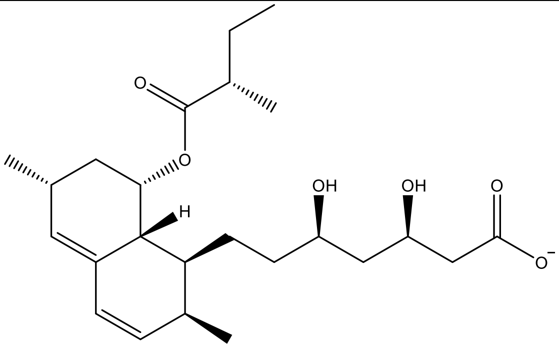<br>421	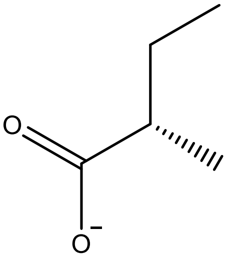<br>101
Pitavastatin[3]	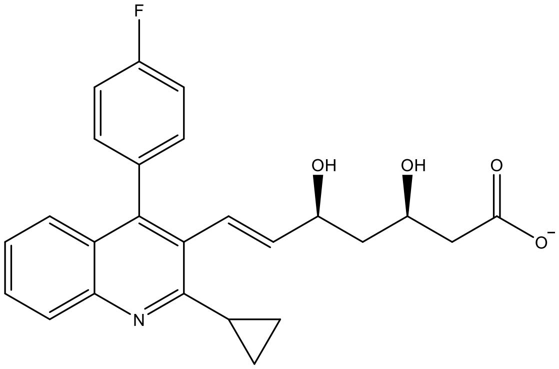<br>420	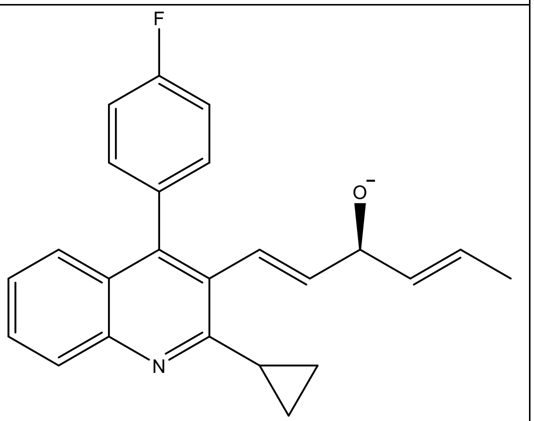<br>358

Pravastatin, Scheme 1[4]	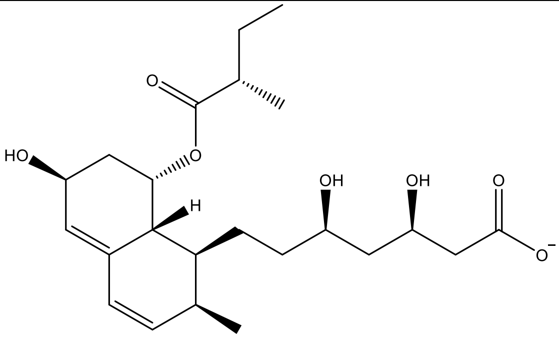 423	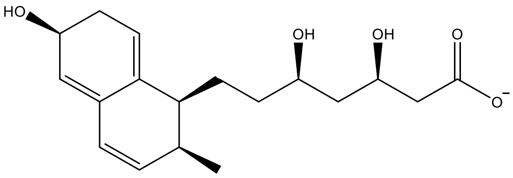 321
Pravastatin, Scheme 2[4]	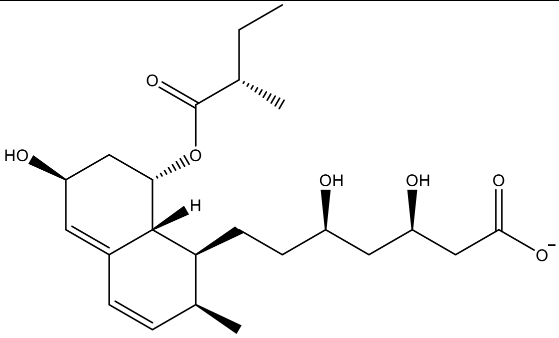 423	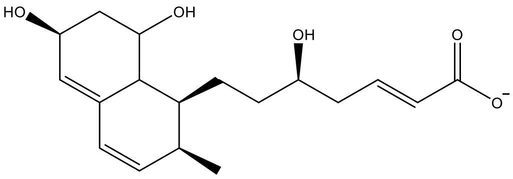 321
Rosuvastatin[3]	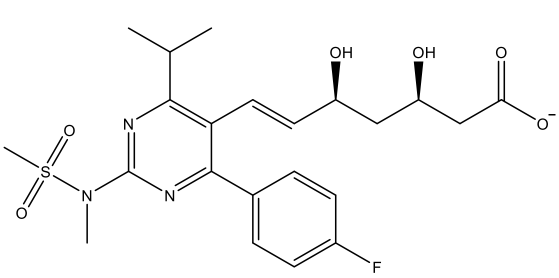 480	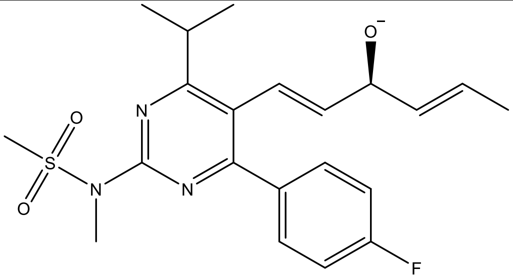 418
Simvastatin Acid, Scheme 1[4]	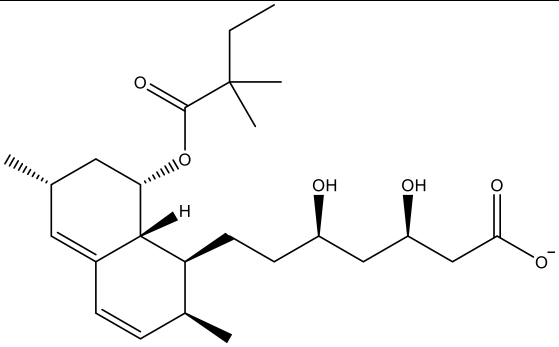 435	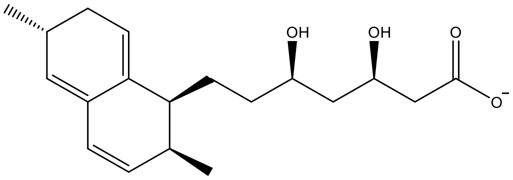 319

Simvastatin Acid, Scheme 2[4]	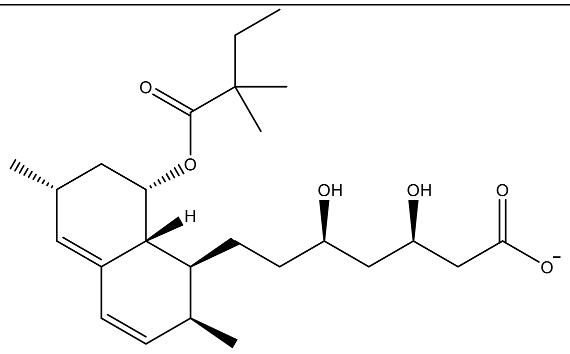 435	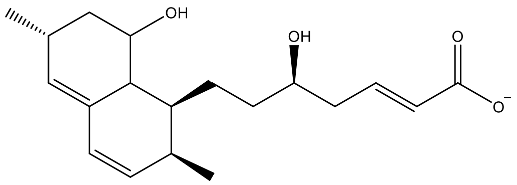 319
N-desmethyl-Rosuvastatin	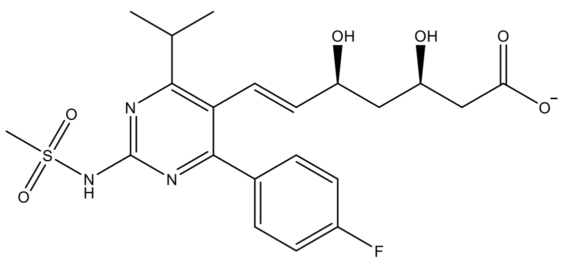 466	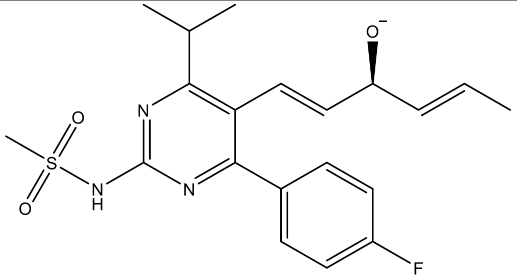 404

Figure S1. Target precursor and fragment products as analyzed by SMRM.

[1]Inferred from atorvastatin fragmentation.
[2] Kant Shandilya, D. , Israni, R. and Edward Joseph, P. (2018) Prediction of the Fragmentation Pathway of Atorvastatin De-Protonated Ion. *Open Access Library Journal*, 5, 1-14. doi: 10.4236/oalib.1104547.
[3] Proposed by us, and confirmed by labeled IS. Not definitive.
[4]Qin, X.-Z. (2003), Collision-induced dissociation of the negative ions of simvastatin hydroxy acid and related species. *J. Mass Spectrom*., 38: 677-686. https://doi.org/10.1002/jms.482

a)

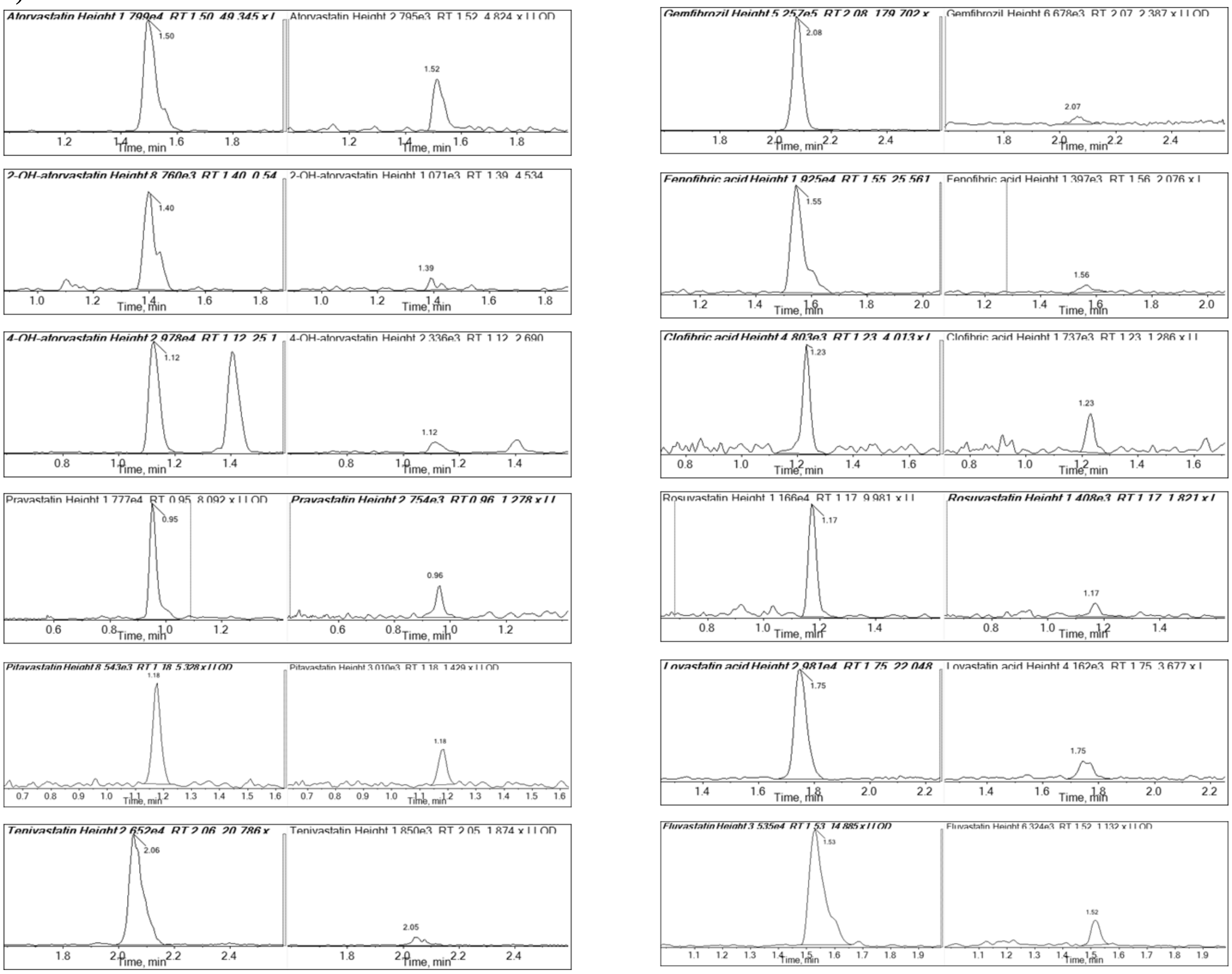

b)

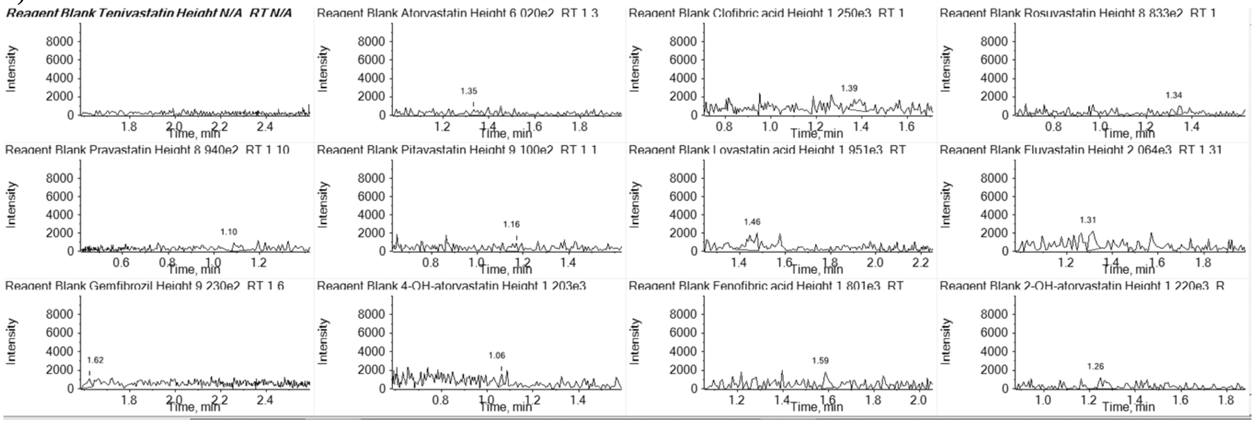

Figure S2. Representative Chromatograms. Individual analyte chromatograms a) at near the median concentrations in plasma samples and near the 5 x LLOD level concentrations and b) blanks.

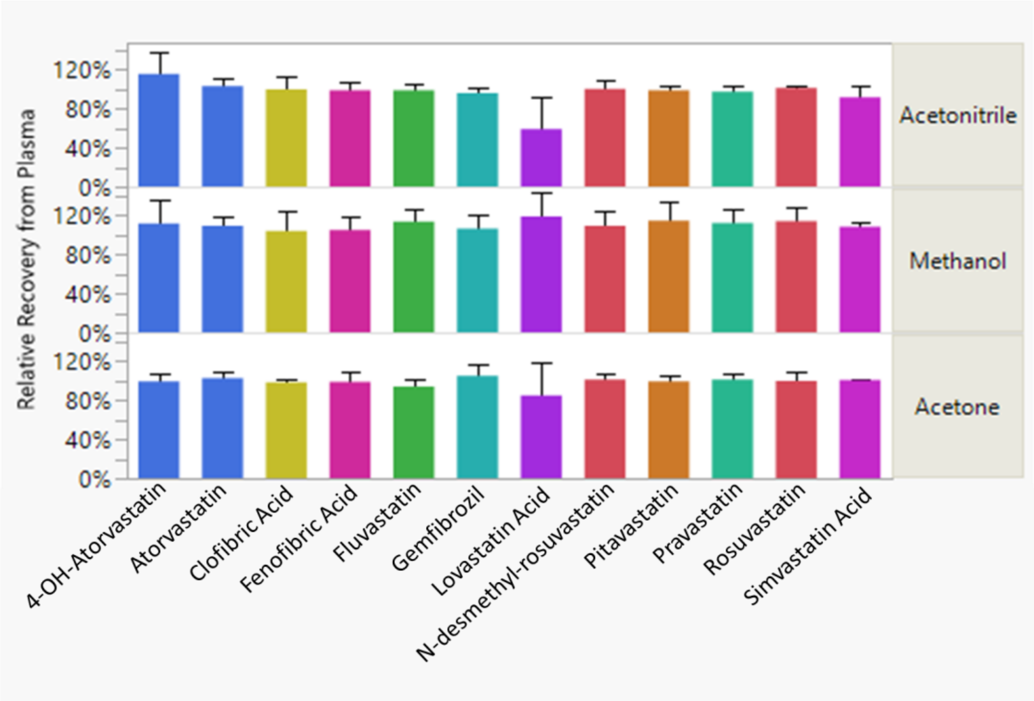

Figure S3. Comparison of Precipitation Solvents for Analyte Recovery. Comparison of protein precipitation solvents by analyte recovery from spiked plasma relative to spiked water. Error bars represent standard deviation of repeats (n = 5). Acetone provided sufficient recovery with greater confidence in measurements and consistency in recovery.

a)

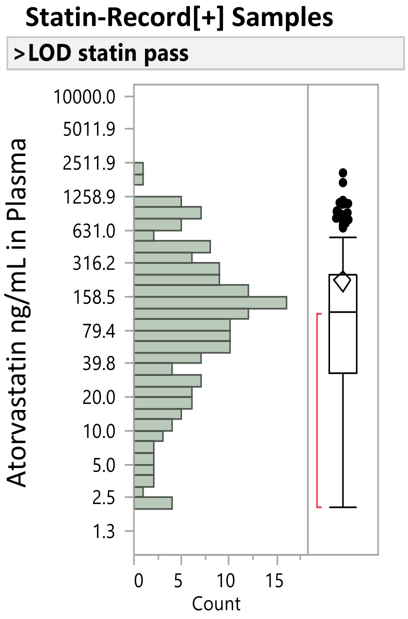

Quantiles

100.0%	maximum	2041.4798117
99.5%		2041.4798117
97.5%		1096.0787895
90.0%		700.04026364
75.0%	quartile	251.92554514
50.0%	median	115.55633057
25.0%	quartile	32.75829861
10.0%		12.05626318
2.5%		2.669865132
0.5%		2.0582938403
0.0%	minimum	2.0582938403

Summary Statistics

Mean	221.65216
Std Dev	314.38718
Std Err Mean	23.564315
Upper 95% Mean	268.15533
Lower 95% Mean	175.149
N	178

b)

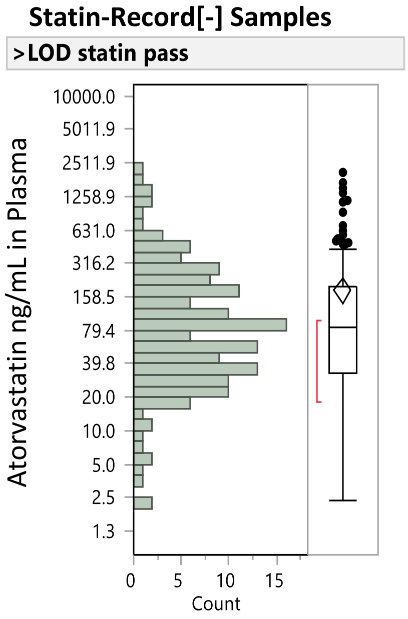

Quantiles

100.0%	maximum	2060.79608
99.5%		2060.79608
97.5%		1346.133218
90.0%		464.17862875
75.0%	quartile	194.6428209
50.0%	median	85.551430938
25.0%	quartile	32.880762156
10.0%		19.057826446
2.5%		4.5606239348
0.5%		2.3661016825
0.0%	minimum	2.3661016825

Summary Statistics

Mean	183.01249
Std Dev	302.52008
Std Err Mean	23.916313
Upper 95% Mean	230.24712
Lower 95% Mean	135.77787
N	160

Figure S4. Distribution Plot of Atorvastatin Detects. Frequency distribution of detections for Atorvastatin Statin-Record [+] (a) and [-] (b) sample groups.

a) b)

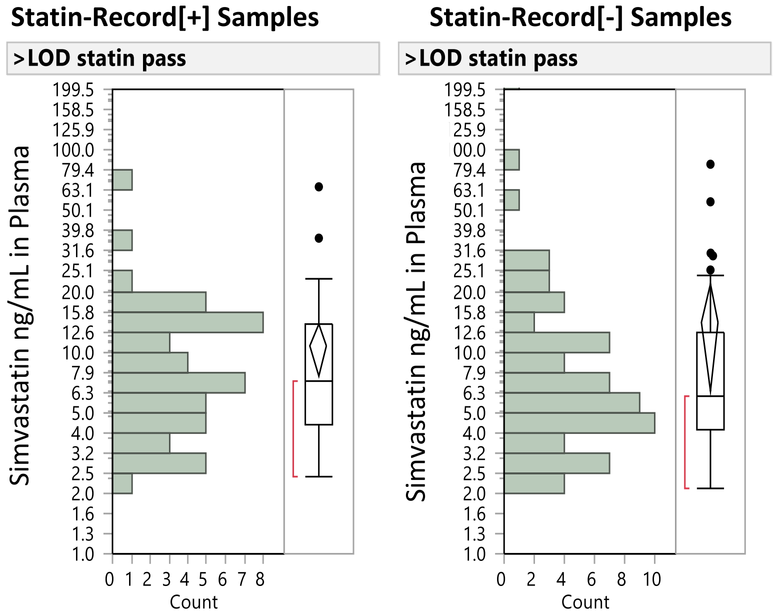

Quantiles (a)

100.0%	maximum	65.767378998
99.5%		65.767378998
97.5%		58.478579367
90.0%		19.003827716
75.0%	quartile	13.805170435
50.0%	median	7.2088402173
25.0%	quartile	4.3414621382
10.0%		2.8687917842
2.5%		2.4438708351
0.5%		2.4210405266
0.0%	minimum	2.4210405266

Summary Statistics (a)

Mean	10.620438
Std Dev	10.418513
Std Err Mean	1.4883589
Upper 95% Mean	13.612985
Lower 95% Mean	7.6278921
N	49

Quantiles (b)

100.0%	maximum	245.32315828
99.5%		245.32315828
97.5%		133.16155255
90.0%		24.090744594
75.0%	quartile	12.372589041
50.0%	median	6.0720260818
25.0%	quartile	4.1113402619
10.0%		2.704371473
2.5%		2.2189396485
0.5%		2.1211808125
0.0%	minimum	2.1211808125

Summary Statistics (b)

Mean	14.019188
Std Dev	31.408554
Std Err Mean	3.8371655
Upper 95% Mean	21.680336
Lower 95% Mean	6.3580399
N	67

Figure S5. Distribution Plot of Simvastatin Detections. Frequency distribution of analytical detections for Simvastatin Statin-Record [+] (a) and [-] (b) sample groups.

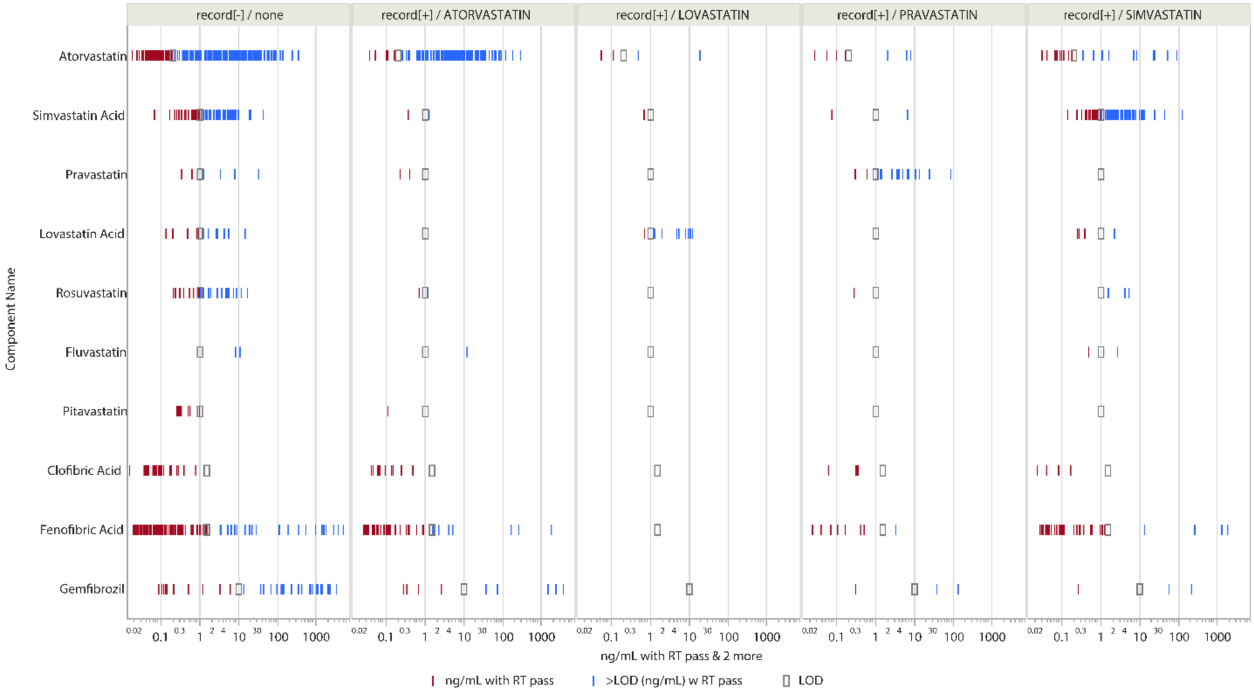

Figure S6. Comparison of Analyte Concentration in Statin-Record[-] and Statin-Record[+]. Results of analysis for all samples with retention time criteria pass groups; the LOD levels are indicated by a small rectangle marker, above LOD level detect is shown with blue lines and below LOD level detect with red lines.

a)
b)
c)

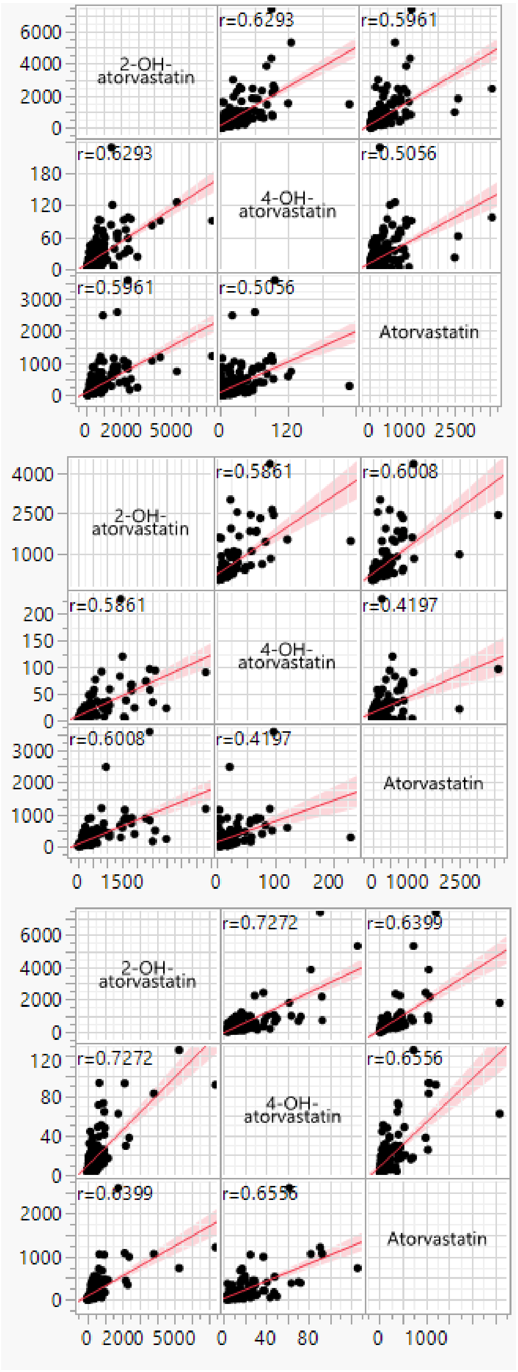

Figure S7: Correlation between Atorvastatin and its Metabolites. a) All samples; b) Statin-Record[-] samples c): Statin-Record[+] samples. All correlations had p-value < 0.0001.
